# Manipulating self and other schemas to explore psychological processes associated with paranoid beliefs: an online experimental study

**DOI:** 10.3389/fpsyg.2024.1474562

**Published:** 2025-01-16

**Authors:** Anton P. Martinez, Elizabeth Milne, Georgina Rowse, Richard P. Bentall

**Affiliations:** Department of Psychology, The University of Sheffield, Sheffield, United Kingdom

**Keywords:** relational schemas, trustworthiness, attachment styles, paranoia, classical conditioning, self-esteem

## Abstract

**Background:**

Information about the self and others is organized in cognitive-affective structures that influence and guide interpersonal behavior. These structures are referred to as relational schemas and are thought to be influenced by early interpersonal experiences with significant others leading to secure or insecure attachment patterns as adults. When insecure, these patterns appear to contribute to paranoid interpretations about the intentions of others by indirect pathways such as negative self-esteem and a bias toward untrustworthiness. Experimental studies employing classical conditioning (CC) interventions have been successful in manipulating these schemas, finding significant effects on various psychological outcomes such as attachment styles, implicit self-esteem, and paranoid beliefs. However, no study to date has explored these effects on trustworthiness judgments.

**Objective:**

This study aims to replicate the findings from previous experiments and also testing the effect of manipulating relational schemas on trustworthiness evaluations.

**Methods:**

A convenience online sample of 266 participants completed a series of tasks and questionnaires measuring attachment styles, explicit and implicit self-esteem, paranoia, and trustworthiness evaluations before and after a brief CC intervention, which involved being randomly allocated to three conditions. In each of these conditions, information about the self was always paired with either positive face stimuli (proximity-seeking condition), negative face stimuli (self-threat condition), or neutral face stimuli (control condition).

**Results:**

This study failed to replicate findings as previously reported in published experiments (i.e., self-esteem, paranoia), only finding a significant effect on attachment styles on the proximity-seeking CC condition. Moreover, no effect was found regarding trustworthiness judgments.

**Discussion:**

Limitations such as the online nature of the study and methodological aspects are discussed.

## Introduction

Paranoia is typically defined as the unfounded belief that other people, groups or organizations are intentionally trying to harm the individual who holds this belief, often manifesting as general suspicion of other’s intentions, interpersonal sensitivity and hypervigilant behaviors (Bentall et al., 2001; Freeman, 2016). Although paranoia is the most common type of delusion reported in adult clinical populations (Collin et al., 2023), it can also be experienced in less severe forms by the general public (Freeman et al., 2007), being better conceptualized as a continuum rather than a discrete category. Several psychological models have been proposed to understand and inform the treatment of paranoid beliefs. These models range from conceptualizing paranoia as result of a discrepancy between an ‘actual’ and ‘ideal’ self (Bentall et al., 1991, 1994), distorted cognitive interpretations fuelled by a vulnerable self (Freeman et al., 2002), or as the product of early adverse attachment experiences (Berry et al., 2008). Although the evidence supporting each model varies, the role of variables such as negative self-esteem and insecure attachment styles on the development of paranoid beliefs is firmly established in the research literature (Murphy et al., 2018; Sood et al., 2022). Moreover, in recent years, there has been more emphasis in highlighting the specific role of mistrust and its association with paranoia in both clinical and non-clinical populations (Kirk et al., 2013; Martinez et al., 2022; Bell and O’Driscoll, 2018; Pinkham et al., 2016). Thus, given the interpersonal nature of paranoid beliefs, it is important to consider a framework that accounts for how cognitive structures about the self and others influence the aforementioned variables in relation to paranoia.

### Relationship schemas: self and other

Social psychology researchers conceptualize relational schemas as cognitive-affective structures that, based on experiences of social interactions, organize patterns of interpersonal behavior, and guide social information processing (Baldwin, 1992). These cognitive structures can be categorized into self and other schemas that reflect beliefs and expectations about oneself and other people, respectively, (Brunson et al., 2015). It is thought that the content of these schemas is organized in associative networks that can be activated when primed with related information (Baldwin, 2005). For instance, studies have shown that participants with negative views of the self who have been presented with self-flaw cues display an attentional vigilance bias toward social rejection stimuli (Ravary and Baldwin, 2018). Behavioral psychologists argue that self-negative information becomes more accessible due to recurrent paring of aversive experiences with social stimuli (i.e., self, other) creating an association that facilitates fearful responses when related cues are presented (Lissek et al., 2008). For example, being repeatedly victimized by peers during adolescence can lead to a self-concept of worthlessness and a view of others as powerful which can elicit hypervigilant behaviors as an adult when faced with similar social situations (Bentall et al., 2012; Stapinski et al., 2014).

The construct of relational schemas is similar to the concept of working models proposed by attachment theorists. However, while the former reflects general knowledge structures about the self, others, and expectations of interpersonal experiences, the latter describes schemas that are activated in attachment-related situations (Dewitte and De Houwer, 2011; Brunson et al., 2015). Hence, attachment styles function as a system that is triggered when faced with a threatening environment with the goal of achieving a general sense of safety (Baldwin and Kay, 2003). The availability of a secure base during childhood will depend on the ability of the primary caregiver (i.e., attachment figure) to successfully meet the emotional needs of the child. If those needs are met, the child is likely to develop a secure attachment style as an adult while, if they are not, an insecure style is more probable (Mikulincer, 1995). People with an insecure-anxious attachment style tend to manifest an excessive longing for closeness as a consequence of their attachment figures being inconsistent in early developmental stages. Conversely, a person with an insecure-avoidant style will deactivate their attachment system, exhibiting extreme distancing from close relationships, reflecting an irresponsive attachment figure during childhood (Dewitte et al., 2007; Dewitte and De Houwer, 2011). Furthermore, these insecure styles are characterized by specific relational schemas. Whereas anxious styles are characterized by having a negative self-schema and a positive other-schema, avoidant styles display the opposite, a positive self-schema and negative other-schema (Dewitte and De Houwer, 2011). Therefore, attachment-working models serve to regulate distress in threatening interpersonal situations and are, in turn, influenced by specific relational schemas (Shaver and Mikulincer, 2005).

Perceptions of social threat and feelings of interpersonal distress stem from a primal human need that dates from early evolutionary times, the need to belong and feel accepted (Baumeister and Leary, 1995). Given that living in groups was crucial for survival, being ostracized or rejected would have diminished the chances to seek food, defend oneself or reproduce, leading to certain death and preventing genes from being passed to further generations (Fiske and Taylor, 2017). Within this framework, social and personality psychologists have argued that self-esteem serves as a *sociometer* that monitors the degree of one’s acceptance and connection with others and directs screening for environmental signals related to socio-evaluative concerns (Leary, 2005; Howell et al., 2019). For example, experimental studies have shown that inducing feelings of acceptance results in increased levels of state self-esteem (Blackhart et al., 2009). Conversely, low-trait self-esteem seems to enhance sensitivity to social cues whereas high-trait self-esteem appears to mitigate the effects of negative social evaluations (Howell et al., 2019). Thus, self-esteem can be conceptualized as a general sense of personal worth that facilitates access to relational schemas representing expectations about social approval or disapproval (Baldwin and Kay, 2003).

Secure early attachment experiences involve warmth and nurturing provided by attachment figures, and these initial interpersonal relations, therefore, set the basis for high or low levels of self-esteem (Hart et al., 2005). Being regarded as competent and worthy of affection by significant others during childhood and adolescence can have enduring psychological effects on a person’s sense of worth throughout their lifespan (Sroufe, 2005). For example, clinical researchers have found that the association between insecure attachment styles (particularly anxious) and depressive symptoms is explained by negative self-esteem (Roberts et al., 1996; Lee and Hankin, 2009). Similarly, the same mediation pathway has been shown to explain the association between insecure attachment and paranoid beliefs (Humphrey et al., 2021; Sood et al., 2022). Moreover, a recent study revealed that although negative self-esteem explained the relationship between anxious attachment and paranoia, a bias toward mistrust was found to explain the association between both attachment styles (avoidant and anxious) and paranoid beliefs (Martinez et al., 2020). Trust is regarded as a core component in relational schemas, particularly in early attachment relations, as it involves positive expectations that significant others would be available in fulfilling one’s emotional needs (Mikulincer, 1998). Hence, by having secure relational schemas one can form automatic impressions that others are trustworthy, easing social interactions with unknown individuals (Todorov, 2008). Conversely, feeling vulnerable and having negative expectations about interpersonal relations can lead to rapid mistrust judgments of unfamiliar faces, enabling avoidance of potentially dangerous strangers but at the same time facilitating hostile interpretations of other people’s intentions (i.e., paranoia; Martinez et al., 2020).

### Experimental paradigms

Several studies have tried to manipulate relational schemas in order to test whether there is an effect in the abovementioned psychological processes (i.e., self-esteem, attachment, paranoia). For example, using a classical conditioning (CC) paradigm Baccus et al. (2004) found that participants whose self-relevant information (e.g., name, date of birth) was paired with smiling faces exhibited higher implicit (but not explicit) self-esteem than those participants whose self-relevant information was paired with random facial expressions. Espinosa et al. (2018) replicated this finding in a student sample with subclinical levels of paranoia with the added effect of showing that the experimental manipulation also lowered subclinical positive symptoms (i.e., unusual experiences) although paranoia levels were unaffected. However, using the same paradigm but with an added negative condition (self-relevant information paired with angry faces), Trucharte et al. (2024) found that student participants in that group reported higher levels of state paranoia. Conversely, participants in a positive condition (self-relevant information paired with happy faces) reported a reduction in interpersonal sensitivity as well as in state anxious and avoidant attachment insecurity. In summary, by carrying out different manipulations with regards to relational schemas, various mechanisms seem to be triggered. Warm associations with self-relevant information seem to activate positive relational schemas and a sense of security, whereas hostile associations elicit negative relational schemas and hypervigilant states.

Some authors have theorized that, in self-threatening situations, individuals experiencing paranoia might adopt a defensive attitude as reflected in a discrepancy between implicit and explicit self-esteem (Bentall et al., 2001; Jordan et al., 2003). According to this kind of defensive model, it might be predicted that pairing self-relevant information with threatening face images will elicit an increase in explicit state self-esteem but, at the same time, a reduction in implicit self-esteem. Nonetheless, although explicit self-esteem seems to be unaffected by a positive CC intervention (Baccus et al., 2004; Espinosa et al., 2018) the effect of negative CC conditions on explicit self-esteem has not been studied.

To date few studies have employed associative or priming interventions to test the effect of relational schemas on trustworthiness judgments. One study found that clinical participants with high paranoia levels in comparison to non-clinical controls rated neutral faces as more untrustworthy when primed with negatively valenced images (Hooker et al., 2011). Although no relational schema primes were used, the study provided evidence that trustworthiness judgments in clinical samples can be influenced by exposure to emotionally negative cues.

### Purpose of the current study

The current study aims to replicate Baccus, Espinosa and Trucharte’s findings and to extend them to include the role of mistrust and paranoia, as highlighted by Martinez et al.’s (2020). Moreover, we aim to explore the specificity of relationship schemas using a CC manipulation on the dependent variables of this study. We employed an experimental design in which participants were randomly assigned to either a self-threat, proximity-seeking, or control condition. In the self-threat condition, self-relevant information was paired with threatening faces, while in the proximity-seeking condition, self-relevant information was paired with non-threatening (i.e., likeable) faces. The control condition involved pairing self-relevant information with random threatening, neutral and likeable faces. The study explored the effect of these conditions on implicit and explicit self-esteem, state paranoia, and state attachment variables. Additionally, trustworthiness judgments were operationalized using an affective priming task, which utilized relational schemas (self and other relevant information) as primes, and previously validated trustworthy and untrustworthy faces as targets. Trustworthiness judgments were analyzed using signal detection analysis to calculate bias scores (i.e., the tendency to judge an untrustworthy face as trustworthy or vice versa).

Based on the aforementioned design we expected the following results:

1. Following the CC intervention, participants in the self-threat condition will show higher levels of explicit self-esteem but lower levels of implicit self-esteem compared to the proximity-seeking and control conditions. Conversely, participants in the proximity-seeking condition, will show higher implicit self-esteem levels compared to the self-threat and control conditions.
2. Following the CC intervention, participants in the self-threat condition will show an increase in state paranoia compared to both the control and proximity-seeking conditions.
3. Following the CC intervention, participants in the proximity-seeking condition will show lower levels of state attachment insecurity (i.e., avoidant, and anxious) compared to the control condition, while higher levels of state attachment insecurity are expected in the self-threat condition compared to the proximity-seeking and control conditions.
4. Following the CC intervention, participants in the self-threat condition will show a bias toward mistrust following a self-relevant prime, but not when presented with an other-relevant prime compared to the proximity-seeking and control conditions. Conversely, participants in the proximity-seeking condition will show a bias toward trustworthiness when presented with a self-relevant prime, but not following an other-relevant prime compared to the self-threat and control conditions.

## Materials and methods

Ethical approval was granted by the Department of Psychology Research Ethics Committee at The University of Sheffield (Ref: 041111).

### Participants

A statistical power analysis was performed for sample size estimation, based on data from a pilot study (*N* = 90). The effect size (ES) in this study for behavioral measures was *η*_p_^2^ = 0.03, considered to be small using Cohen’s (1992) criteria. With an alpha = 0.05 and power = 0.95, the projected sample size needed with this effect size (GPower 3.1) is approximately *n* = 252 for the simplest between/within-group comparison. Thus, our proposed sample size of *n* = 307 was more than adequate for the main objective of this study and allowed for expected attrition and our additional objectives of controlling for possible factors/subgroup analysis.

Participants were recruited via social media platforms (e.g., Twitter) as well as the volunteer staff list from the University of Sheffield and were offered a £5 AMAZON voucher once they have completed the study. The ages of the participants ranged from 18 to 73 (*M*_age_ = 28.9, *SD* = 7.7), and the sample was characterized by being majority male (51.9%), highly educated (59.4% graduates), and employed (62%). From 307 respondents, 266 were finally selected after excluding participants who did not pass more than 50% (3) of attention checks (e.g., “*Please select option number six*”) as well as those who were considered extreme outliers of survey completion time based on Mahalanobis D (Curran, 2016). Excluded participants did not differ from those who were included in any of the demographics or psychological variables (*p* > 0.05).

### Measurements

The revised Paranoia and Deservedness Scale (PaDS–R; Elahi et al., 2017): the PaDS is a paranoia trait measure validated in clinical and non-clinical populations that consists of 10 items which are answered on a 5-point scale from 0 (Certainly False) to 4 (Certainly True) with total scores ranging from 0 to 40. This instrument in turn has two scales, a persecution one that measures paranoid ideation and a deservedness one that assesses the degree to which respondents feel they deserve what is described in each persecution item. For this study, only the persecution scale was used reflecting good reliability (*α* = 0.77).

The Relationship Questionnaire (RQ; Bartholomew and Horowitz, 1991). The RQ is a self-report instrument that describes in four short paragraphs secure, fearful, preoccupied, and dismissing attachment patterns. By reading each description participants rate how well or poorly each vignette defines their corresponding relationship pattern on a 7-point scale ranging from 1(Disagree strongly) to 7 (Agree strongly). The scoring of each scale serves to compute measures of insecure styles such as attachment anxiety (negative model of self) and attachment avoidance (negative model of other)^1^. Negative scores will indicate the presence of insecure models for each attachment representation whereas positive scores will reflect the opposite. Reliability analysis using Cronbach’s *α* cannot be calculated because there is only one item per attachment type, but good psychometric properties such as test–retest, construct, convergent and divergent validity for this scale have been established (Wongpakaran et al., 2021).

Rosenberg Self-Esteem Scale (RSES; Rosenberg, 1965): The positive subscale comprises 10 items designed to measure trait positive self-esteem by asking participants to rate on a 4-point Likert scale each statement from 1 (Strongly Agree) to 4 (Strongly Disagree). Total scores range between 10 to 40 and reliability analyses revealed acceptable levels (*α* = 0.63).

Name letter preference task (NLT; Nuttin, 1985): the NLT is an implicit self-esteem measure that consists of asking participants to rate their liking of the letters of the alphabet from 1 (Not at all) to 10 (A lot). The rationale behind this task lies in the premise that people with high implicit self-esteem tend to rate more positively the letters of their own names over other letters of the alphabet. A recommended algorithm for calculating implicit self-esteem scores is the *ipsatized* double-correction algorithm that controls for differences in the likeability of the different letters as well as the frequency of more generally used letters (LeBel and Bertram, 2009). The NLT has shown good levels of internal validity (*α* = 0.83) and test re-test reliability (Krause et al., 2011).

State Self-Esteem Scale (SSES; Heatherton and Polivy, 1991): the SSES is a 21-item scale designed to assess momentary states of self-esteem. Responses are provided on a 5-point Likert scale (1 = not at all, 2 = a little bit, 3 = somewhat, 4 = very much, and 5 = extremely). This scale has shown good internal reliability (*α* = 0.75 to *α* = 0.80) and good construct validity with total scores varying between 10 and 105.

State Adult Attachment Measure (SAAM; Gillath et al., 2009): is a scale developed to assess temporary states of insecure (anxious, avoidant) and secure attachment in response to experimental manipulations. The scale includes 21 items where participants have to rate the extent to which they agree or disagree with different statements based on how they currently feel from 0 (Disagree strongly) to 7 (Agree strongly). For this study, we only used the insecure attachment subscales (7 items per subscale) with total scores ranging from 0 to 49. The SAAM has shown good internal reliability (*α* = 0.83–0.87), discriminant, convergent, and criterion validity.

State Paranoia Checklist (SPC; Schlier et al., 2016): is an 18-item scale designed to assess mild persecutory ideas. This version of the SPC has been adapted to measure state paranoid beliefs by asking participants to what extent each item applies to them “at the moment.” Answers are provided on a Likert scale that ranges from 0 (not at all) to 10 (very much) with total scores varying from 0 to 180. The SPC has excellent internal reliability (*α* = 0.96) and good convergent validity (Lincoln et al., 2013).

**Figure 1:**
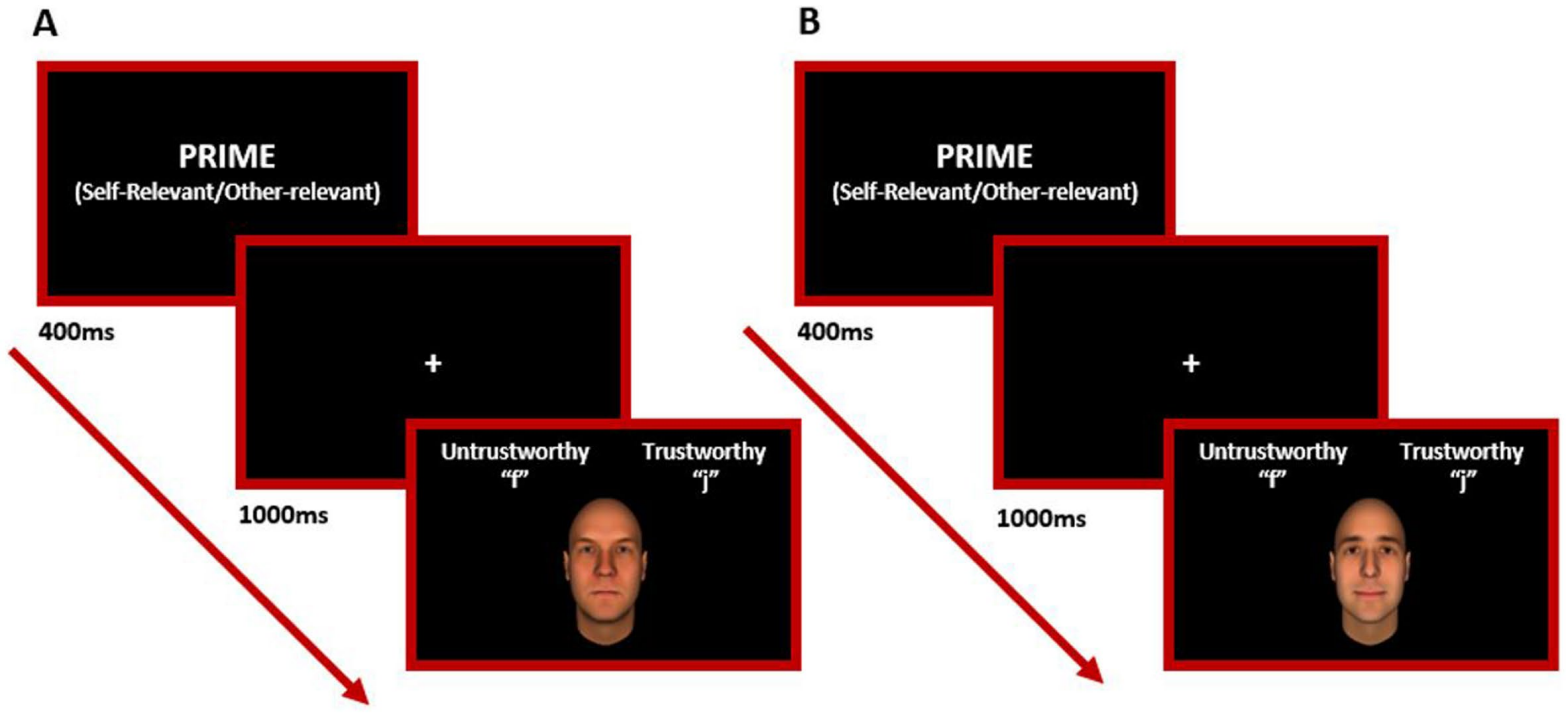
Flow trial diagram for untrustworthy **(A)** and trustworthy **(B)** trials.

Affective priming task (APT; Fazio et al., 1986): the APT is an evaluation task in which participants categorize a target stimulus in a binary way (e.g., positive or negative image) after being primed with a valenced stimulus (e.g., positive or negative word). The rationale behind this task is that if the prime triggers the same response as the target the response is facilitated, reflected in lower error rates. However, if the prime and target are incongruent the response becomes conflicted leading to a higher error rate. In this study, an adaptation of this task was conducted by presenting self-relevant and other-relevant information as primes and trustworthy and untrustworthy face stimuli as targets. For this, computer-generated faces from the Princeton Social Perception Lab dataset (Oosterhof and Todorov, 2008) previously calibrated in the trustworthiness dimension as either more trustworthy (+3 and +2 SD) or less trustworthy (−3 and −2 SD) were used as target stimuli (Supplementary Figure S1). Word stimuli (personal information and non-personal information) provided for the conditioning paradigm were used as prime stimuli. The structure of the task comprised 144 trials of which two blocks of 72 trials involved trustworthy and untrustworthy presentations of targets, respectively. Moreover, of those 72 trials, 36 trials included “other” primes whereas the remaining 36 included “self” primes. Finally, each block of 36 trials encompassed 12 trials for three different types of face ethnicity (White, Black, and Southeast Asian). For each trial, the prime word was displayed for 400 ms followed by a central fixation point (+) that was presented for 1,000 ms and the target image (e.g., trustworthy or untrustworthy face) appeared immediately afterward and remained on the screen until participants made a response by pressing the “j” (trustworthy) or “f” (untrustworthy) key (see Figure 1). One hundred and forty-four trials in total were randomized for each participant to control for an order effect. Participants completed six practice trials with neutral words before commencing with the actual task.

### Procedure

To assess the causal effect of self-threat and proximity-seeking on trustworthiness judgments, implicit self-esteem, and state measures (attachment, paranoia, and self-esteem), the study followed an experimental between/within-subjects design. Participants were randomly assigned either to the experimental conditions (self-threat, proximity-seeking) or the control condition (neutral) and completed outcome measurements before and after the experimental manipulation. Participants who took part in the study had to read the participant information sheet and consent. Once they agreed on participating, they were asked to answer self-report questionnaires regarding trait measures (attachment styles, paranoia, and self-esteem) and state measures (self-esteem, paranoia, and attachment). They also were asked to complete affective priming tasks on trustworthiness judgments and implicit self-esteem to establish a baseline before manipulation. This pre-measurement baseline phase was established 3 days before the intervention to control for any type of carryover effects that could influence the experimental manipulation. This period between pre- and post-manipulation was based on Dewitte and De Houwer's (2011) findings in which participants were measured 3 days before being primed with specific attachment schemas. For this purpose, participants were asked to enter their email addresses so they could be reminded^2^ to participate in the second part of the study and receive their £5 AMAZON voucher once the whole study was completed.

**Figure 2:**
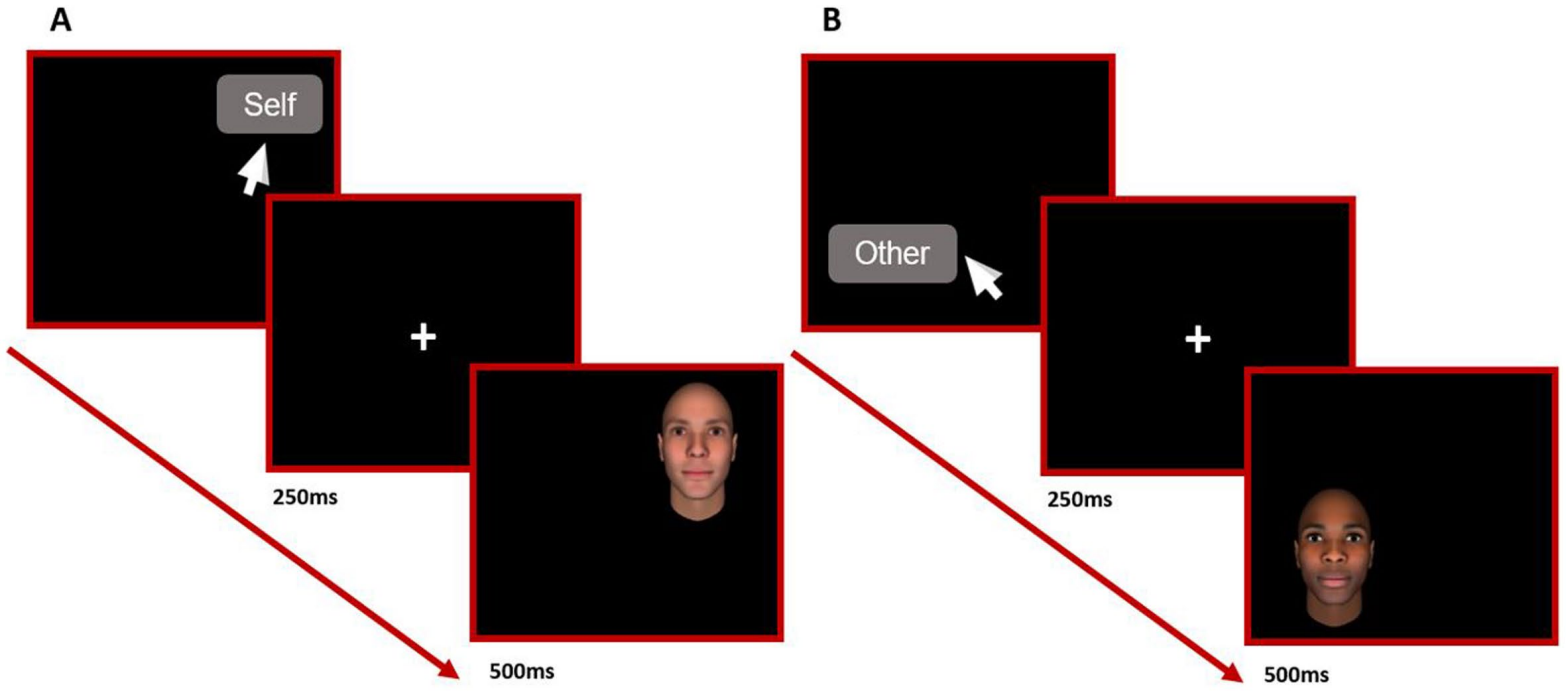
Flow trial diagram for self **(A)** and other **(B)** classical conditioning trials.

The classical conditioning intervention was based on the one implemented by Baccus et al. (2004), which involves asking participants to provide self-relevant information (i.e., first name/nickname, last name, the month and day of birth, personal pronouns me/mine). This information was collected at baseline which was also used for the affective priming task. These words were matched with control words such as names, surnames, and months and days different from the information provided by the participants (see Supplementary Table S1). Participants were randomized to either the experimental (self-threat, proximity-seeking) or control conditions using the balanced randomization mode provided in the Gorilla online experiment builder. For completing the intervention, they were informed that a word would appear randomly in one of the quadrants on the computer screen and they were instructed to click on the word as quickly as possible, using the mouse. In addition, they were told that when they did so an image would be displayed briefly (for 500 ms) in that quadrant preceded by a fixation cross (for 250 ms, Figure 2). This procedure was repeated for 252 trials. Self-relevant words (self-relevant information) and other-relevant words (control words) were presented in a preprogrammed pseudorandom order. In the control condition, once the participant clicked the word stimuli, a random selection of threatening (84 times), non-threatening (84 times), and neutral (84 times) photographs of faces followed both self-relevant and non-self-relevant words. In the experimental self-threat condition, self-relevant words were always paired with an image of a threatening face (126 times) whereas non-self-relevant words were paired with non-threatening (42 times), threatening (42 times), and neutral faces (42 times). In the experimental proximity-seeking condition, self-relevant words were always paired with non-threatening (i.e., likeable) face images (126 times), and non-self-relevant words were paired with neutral (42 times), non-threatening (42 times), and threatening face images (42 times). Face images were also downloaded from the Princeton Social Perception Lab dataset which has been previously validated in the social threatening dimension calibrated as more threatening (+3 and +2 SD) as well as less threatening (−3 and −2 SD; Supplementary Figure S3). These face stimuli also represented different ethnicities (White, Black, and Southeast Asian) which were equally presented in each condition. The experiment was programmed in a way that participants’ self-relevant information did not overlap with the other-relevant information. The task was self-paced with forced responses, so participants could not proceed to the following trial unless they clicked the quadrant in which the word appeared to minimize careless responses.

After the classical conditioning intervention, participants were asked to complete again the state measurements (attachment, self-esteem, and paranoia) as well as the affective priming and implicit self-esteem task administered in the pre-measurement phase. The order in which the measurements were presented, at both pre as well as post-time points, were randomized using *Latin square* mode provided by Gorilla online experiment builder.

### Statistical analysis

Chi-square tests as well as univariate ANOVA were conducted to compare differences at baseline regarding demographics along with psychological variables between groups. For the trustworthiness outcome, a 2 (time) × 3 (ethnicity) × 3 (conditions) repeated measures ANOVA was conducted to control for the effect of ethnicity on trustworthiness judgments. For the rest of the outcomes, a 2 (time) × 3 (conditions) repeated measures ANOVA was conducted. All analyses were conducted in SPSS v28.

For trustworthiness judgments, signal detection analysis was conducted to calculate the bias (*β*) parameter using the formula 7 reported by Stanislaw and Todorov (1999). In this context, a positive value would indicate a tendency to judge an untrustworthy face as trustworthy whereas a negative value would reflect the opposite. This outcome was used to compute a prime index for the affective priming task by subtracting the bias scores of the “self” priming condition from the “other” priming condition (Prime Index = βSelf – βOther). A positive prime index would indicate a positive bias when primed with self-relevant information in comparison to being primed with other-relevant information. Conversely, a negative prime index would reflect a negative bias when primed with self-relevant information in comparison to being primed with other-relevant information (Wentura and Degner, 2010).

## Results

No significant differences were found when comparing demographic and trait psychological variables between the experimental and control groups (Table 1).

**Table 1:** Descriptive statistics.

Variables	Condition			Total *N* = 266	*p*
	Self-Threat *N* = 93	Proximity-Seeking *N* = 81	Control *N* = 92		
Demographics					
Mean age	29.1 (6.9)	28.5 (8.4)	29.0 (7.7)	28.9 (7.7)	0.85
Gender	48.3% (M)	56.7% (M)	51.1% (M)	51.9% (M)	0.53
Education	58.1% (HE)	59.2% (HE)	60.7% (HE)	59.4% (HE)	0.79
Employment	65.6% (E)	58% (E)	61.9% (E)	62% (E)	0.55
Psychological traits					
Paranoia	30.1 (6.1)	28.3 (6.5)	28.3 (6.7)	28.9 (6.4)	0.10
Self-esteem	24.2 (3.7)	23.5 (4.5)	23.7 (4.0)	23.8 (4.1)	0.48
Attachment Avoidance	−0.20 (2.9)	0.05 (3.7)	0.17 (3.1)	0.003 (3.2)	0.72
Attachment anxiety	0.50 (3.9)	1.1 (4.0)	0.45 (4.0)	0.66 (3.7)	0.50

SD in brackets for numerical variables. M, male; HE, higher education (undergraduate degree or higher); E, employed.

For the rest of the mixed repeated measures ANOVA, normality and homogeneity of variance assumptions were checked by using visual inspections (Q–Q plots and histograms, see Supplementary Figures S1, S2) and Levene’s test, respectively. Levene’s tests revealed non-significant results for all variables (Supplementary Table S2) meaning that error variances were equal across groups.

Regarding normality, all variables seem to display normal distributions of their residuals except for the state paranoia variables in which their distribution seemed to be moderately negatively skewed (−0.72). Transformations of non-normal distributions as well as outliers’ corrections were based on the recommendations detailed in Tabachnick et al. (2013).^3^ Sphericity assumption was checked for the first analysis as ethnicity had more than two within-subject levels (White, Black, and Southeast Asian) unlike time which only had two (pre and post measures). Mauchly’s test of sphericity revealed non-significant results [*χ*^2^(2) = 0.99, *p* = 0.20] indicating that this assumption was not violated.

Regarding implicit self-esteem as measured by the name letter task, no significant effect of time was found [*F*(1,263) = 3.29, *p* = 0.07, *η*_p_^2^ = 0.012], nor interaction effect between time and condition [*F*(2,263) = 0.23, *p* = 0.80, *η*_p_^2^ = 0.002]. The same non-significant results were found on state self-esteem for the effect of time [*F*(1,263) = 0.3.01, *p* = 0.08, *η*_p_^2^ = 0.01], and the interaction effect between time and condition [*F*(2,263) = 0.460, *p* = 0.63, *η*_p_^2^ = 0.003]. Thus, hypothesis 1 was not supported in this study. Moreover, hypothesis 2 was also not supported, since analyses concerning state paranoia yielded non-significant results for the effect of time [*F*(1,263) = 2.00, *p* = 0.16, *η*_p_^2^ = 0.008], and the interaction effect between time and condition [*F*(2,263) = 0.1.06, *p* = 0.35, *η*_p_^2^ = 0.008].

A significant main effect of time was found for state attachment avoidant style [*F*(1,263) = 4.27, *p* = 0.04, *η*_p_^2^ = 0.02] revealing a reduction of attachment-avoidant scores from pre (*M*_T1_ *=* 26.98) to post (*M*_T2_ *=* 26.35) experimental manipulation. Nonetheless, this change was not moderated by experimental conditions as the interaction effect between time and condition was not significant [*F*(2,263) = 0.61, *p* = 0.54, *η*_p_^2^ = 0.005]. In the case of state attachment anxiety, a main effect of time was also found [*F*(1,263) = 13.80, *p* < 0.001, *η*_p_^2^ = 0.05] revealing a significant decrease of state attachment-anxiety levels from pre (*M*_T1_ = 30.28) to post (*M*_T2_ = 29.10) intervention. Moreover, results revealed a significant interaction effect between time and condition [*F*(2,263) = 3.00, *p* = 0.05, *η*_p_^2^ = 0.02]. Pairwise comparisons with Bonferroni corrections showed that participants in the Proximity-Seeking condition reported lower levels of insecure attachment levels post-intervention (*M*_T1_ = 30.90, *M*_T2_ = 28.66) in comparison to the control and Self-Threat condition in which no changes were found, partially supporting hypothesis 3 (Table 2).

**Table 2:** Pairwise comparisons.

	State anxious attachment							
Condition	(I) Time	(J) Time	*M_diff_* (I – J)	*SE*	Sig.^a^	*η*_p_^2^	95% CI	
							[LB/UB]	
Control	1	2	0.97	0.54	0.07	0.01	−0.09	2.03
Proximity-seeking	1	2	**2.23**	**0.320**	**<0.001**	0.05	**1.10**	**3.36**
Self-threat	1	2	0.33	0.53	0.53	0.001	−0.72	1.39

Based on estimated marginal means^a^. Adjustment for multiple comparisons: Bonferroni. Bold represents significant values.

With respect to trustworthiness judgments, the main effect of ethnicity on the Prime Index (*β*) [*F*(2,526) = 2.03, *p* = 0.13, *η*_p_^2^ = 0.01], as well as the interaction effect between condition, time, and ethnicity [*F*(2,526) = 1.88, *p* = 0.11, *η*_p_^2^ = 0.01], were non-significant. This would indicate that the ethnicity of the stimuli did not influence trustworthiness judgments regardless of the condition participants were in or the time at which the task was completed. When looking at the interaction between condition and time, no significant main effect of time [*F*(1,263) = 0.16, *p* = 0.70, *η*_p_^2^ = 0.001], nor significant interaction between time and condition [*F*(2,263) = 0.006, *p* = 0.97, *η*_p_^2^ = 0.001] were found.

## Discussion

This study aimed to explore the effect of relational schemas on attachment, self-esteem, trustworthiness judgments, and paranoia processes by pairing self and other-relevant information with likeable, threatening, and neutral face stimuli. For our first hypothesis we expected that, compared to the control condition, participants in the proximity-seeking condition would exhibit higher implicit self-esteem but no change in explicit state self-esteem. For participants in the self-threat condition, we predicted they would report a discrepancy between higher state self-esteem and lower implicit self-esteem reflecting a defensive response to threat. In contrast to what Baccus et al. (2004) and Espinosa et al. (2018) found, implicit self-esteem levels in the proximity-seeking condition did not increase after the intervention. Likewise, a discrepancy between implicit and explicit self-esteem was not found in the self-threat condition after the experimental manipulation. Moreover, in our second hypothesis, we did not find higher state paranoia levels in the self-threat group after the intervention, thereby not replicating Trucharte et al.’s (2024) findings. Nonetheless, our third hypothesis was partially met as a decrease in state avoidant attachment levels was found regardless of the effect of a particular intervention whereas lower state anxious attachment levels were explained by the proximity-seeking condition. In our last hypothesis, we stated that the condition in which participants were would influence trustworthiness judgments. Relative to the control condition, we expected that in the self-threat condition, participants would show a biased response toward mistrust when a self-relevant prime preceded the targets and the opposite (a bias toward trust when primed with self-relevant cues) in the proximity-seeking condition. Results did not support this hypothesis, as when or in which condition participants completed the task did not affect their trustworthiness judgments.

Findings from the first and second hypotheses mirror failed replications from previous studies that used the same experimental paradigm. These null findings, to some extent, could be explained by the fact that previous studies might represent false positive results. Sample sizes in each study ranged from 20 to 28 in clinical and subclinical groups, and from 118 to 160 in non-clinical groups, with most studies showing small to moderate effects, although only one of them reported power calculations (Trucharte et al., 2024). Nonetheless, there are several methodological factors that should be considered when interpreting these results.

First, we conducted the study online due to the COVID-19 pandemic whereas the studies on which we based the replication were conducted in-person. Although online studies pose clear advantages over lab-based experiments such as rapid recruitment and relatively low cost for researchers, many other aspects may compromise the reliability of online responses. For example, not being able to check if the information participants provide is accurate (e.g., incorrect demographics), or if they are trying to profit from the study (e.g., taking part in the study more than once), or not being motivated enough (e.g., careless responses). Given this, several aspects were taken into account when designing the study to ensure methodological quality, such as randomization procedures, implementing attentional checks, controlling for carry-over effects, and financial reward upon completion of the whole study. Nonetheless, accounting for these potential sources of unreliable responses is probably not enough to ensure the completion of the intervention in a controlled and quiet environment, possibly compromising the effectiveness of the experimental procedure.

A second factor that differentiates the current study from the ones upon which we based our replication is the use of face stimuli for the classical conditioning intervention. Whereas the original studies employed face stimuli expressing happy, neutral, and angry expressions from the Karolinska Directed Emotional Faces database, we used computer-generated faces on the threatening–likeable dimension from the Princeton Social Perception Lab database. The decision of employing the latter database was due to certain advantages over face photographs of human actors. For example, controlling for specific features of facial expressions such as different variations of the emotion displayed as well as individual differences exhibited by the actors in their expressions (Said and Todorov, 2011). Although controlling for these aspects could be beneficial for psychophysiological studies, the use of computerized faces might seem more unnatural in contrast to the faces of human actors and thus might not be ideal for activating relational schemas. However, the use of this dataset seemed to have had an effect, albeit small, on state-anxious attachment. Given that an insecure-anxious attachment style is characterized by having a negative self-schema and a positive other-schema, it might not be surprising that pairing likeable faces with self-relevant information would elicit a general sense of security. Another explanation for this finding is that state attachment style is more sensitive to change in comparison to other constructs such as implicit self-esteem and thus it is more easily activated using computerized face stimuli. Future research should focus on the effect that different types of face stimuli (i.e., computer-generated; human faces) can have on eliciting relational schemas and its effect on various related psychological variables (i.e., self-esteem, attachment, paranoia, and trustworthiness).

Finally, trustworthiness judgments while primed with relational schemas were not affected by the classical conditioning intervention. Given the manipulation of relational schemas was not successful it perhaps is unexpected that no impact on trustworthiness judgment was seen. Implementing an evaluative (or classical) conditioning intervention involves establishing an association between an unconditioned stimulus (*US*: self-relevant word) with a conditioned stimulus (*CS*: threatening/likeable face). Thus, we operationalized trustworthiness judgments using an evaluative (affective) priming task so that the learning effect of the conditioning intervention would be reflected in a facilitated response when a prime (*US*) preceded a target (*CS*). An explanation for this null effect could be that the valence of the *CS* was not high enough for the *US* to trigger a conditioned response (Hofmann et al., 2010). Another potential explanation could be the latency of the stimulus onset asynchrony (SOA) used in this study for the affective priming task, being 1,000 ms and thus impeding the facilitation of automatic associations between prime and target. Furthermore, a bias toward mistrust measured as the outcome of an affective priming paradigm at baseline did not reveal significant correlations with paranoid traits nor with attachment styles (Supplementary Table S3) probably indicating weak convergent validity. Previous studies using standard facial recognition tasks with the same stimuli have found significant associations between a bias toward mistrust and paranoia as well as with insecure attachment styles in large international representative samples (Martinez et al., 2020, 2022). Standard and priming emotion recognition paradigms are designed to measure different psychological processes. Whereas the former focuses on explicit processing of emotions, the latter aims to capture nuanced responses elicited by contextual information (Kliemann et al., 2013). Thus, this could lead to differences in observed associations between the same construct measured differently and an outcome. Hence, the fact we did not employ a representative sample in this study or used weak primes in our affective priming task may have led to an unreliable measure of trustworthiness judgments. Future research could benefit from exploring the role of trustworthiness judgments by implementing other experimental manipulations such as social rejection and stress paradigms which have shown to elicit paranoid beliefs in non-clinical populations (Kesting et al., 2013; Lincoln et al., 2013).

### Limitations

As mentioned in the abovementioned paragraphs, this study is not exempt from limitations. First, the use of an online convenience sample does not reflect the characteristics of a representative general population sample. Although the gender distribution was not distal from the one of the general population, the sample in this study was mainly highly educated, young, and employed and hence our results are not generalizable. Moreover, online recruitment was done via social media, and may have attracted careless responders, rather than via well-known survey platforms which tend to guarantee quality respondents. Lastly, although the face stimuli dataset used for both the trustworthiness task as well as for the classical conditioning intervention included three different types of ethnicities, the faces were primarily male thus limiting the generalizability of the results. Though the ethnicity of the faces was controlled for, revealing that did not influence trustworthiness judgments, the ethnicity of the participants was not collected thus constraining our interpretations of this finding.

### Methodological implications

Due to contextual circumstances such as the COVID-19 pandemic, this research was conducted online and thus faced several limitations. Nonetheless, significant, and null findings of this research can lead to a number of methodological implications. First, the same procedures should be followed when replicating experimental paradigms, in the case of the current study real face stimuli as well as in-person data collection may have had an impact on the results. Second, it is possible that, to generate a significant effect on trustworthiness ratings operationalized as an affective priming task, the valence of the *CS* should be powerful enough for the *US* to elicit a response as well as using shorter SOA. Thirdly, when measured using priming or standard face recognition paradigms, trustworthiness judgments might tap into different psychological processes leading to different observed associations between the same construct and other variables. Lastly, insecure state anxious attachment seems to be more sensitive to change meaning that, by activating positive self-relational schemas, insecure attachment state levels are reduced leading to an overall feeling of security.

## Conclusion

This study aimed to experimentally manipulate relational schema to see its effect on different psychological constructs that tap onto social cognitive processes that are thought to be underlying mechanisms of psychological symptoms-traits such as paranoia. Previous studies employed classical conditioning interventions to elicit such processes however, they did not consider trustworthiness judgments, a core component of attachment and paranoid beliefs. Our results only showed a reduction of insecure attachment when pairing positive valenced face stimuli with self-relevant information however, given the abovementioned limitations these findings and conclusions should be interpreted with caution. Future research should replicate these findings by comparing online versus lab-based data collection as well as by implementing different face stimuli datasets to explore the effect of human faces versus computer-generated ones.

## Data Availability

The raw data supporting the conclusions of this article will be made available by the authors, without undue reservation.

## Footnotes

^1^Each style can be calculated as follows: model of self = (secure + dismissing) - (preoccupied + fearful); model of other = (secure + pre-occupied) – (dismissing + fearful).

^2^This was done by using the Gorilla Experiment builder delay node which automatically sent an email with a link to complete the second part of the experiment 72 h after participants finished the first part of the study.

^3^NEWX = SQRT (K-X). Where SQRT refers to squared root transformation and K-X is the reflected variable in which each score has been subtracted from the largest score plus 1 in the distribution.
