# Supplementary material for: Manipulating self and other schemas to explore psychological processes associated with paranoid beliefs: an online experimental study
Source: Front Psychol. 2025 Jan 16;15:1474562. doi: 10.3389/fpsyg.2024.1474562 (PMC11781115; doi:10.3389/fpsyg.2024.1474562)
Supplement: Supplementary file 1 [file Data_Sheet_1.docx]

Supplementary Material

Martinez et al. (2024)

| **Table S1.** *Other-relevant information for classical conditioning task.* | | | | |
| --- | --- | --- | --- | --- |
| Names | Surnames | Dates | Cities | Pronouns |
| *Marvin* | *Dankworth* | *February 29th* | *Berlin* | *He* |
| *Siobhann* |  |  |  | *She* |
| *Ludovic* | *McQoid* | *December 21st* | *Brussels* | *His* |
| *Tatania* |  |  |  | *Her* |

| **Table S2.** *Levene's Test of Equality of Error Variances^a^* | | | | |
| --- | --- | --- | --- | --- |
|  | Levene Statistic | df1 | df2 | Sig. |
| Prime Index (T1) | .91 | 2 | 263 | .40 |
| Prime Index (T2) | .94 | 2 | 263 | .39 |
| State Paranoia (T1) | .75 | 2 | 263 | .47 |
| State Paranoia (T2) | .50 | 2 | 263 | .61 |
| State Self-Esteem (T1) | 1.35 | 2 | 263 | .26 |
| State Self-Esteem (T2) | 1.54 | 2 | 263 | .22 |
| Implicit Self-Esteem (T1) | 2.27 | 2 | 263 | .10 |
| Implicit Self-Esteem (T2) | .14 | 2 | 263 | .87 |
| State Attachment Anxiety (T1) | 1.21. | 2 | 263 | .30 |
| State Attachment Anxiety (T2) | 1.61 | 2 | 263 | .20 |
| State Attachment Avoidance (T1) | .27 | 2 | 263 | .76 |
| State Attachment Avoidance (T2) | 1.80 | 2 | 263 | .17 |
| ^a^Levene’s values based on the means | | | | |

**Figure S1.** *QQ-plots for Dependent Variables at different time point.* **
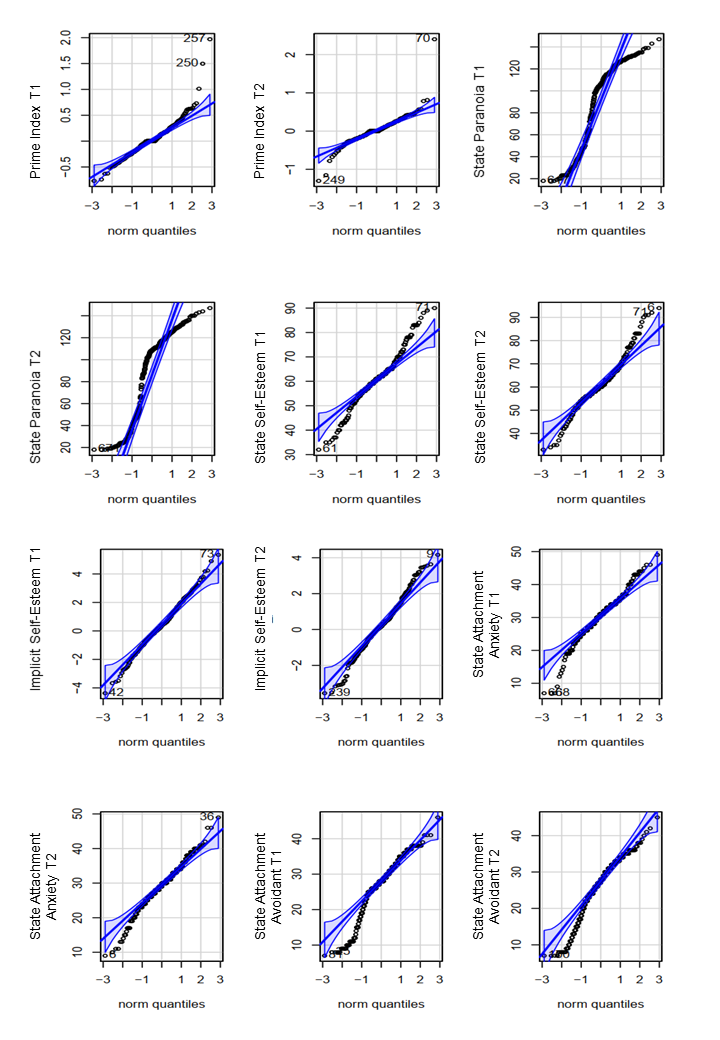
**

**Figure S2.** *Histograms of residuals for Dependent Variables at different time points.*

**
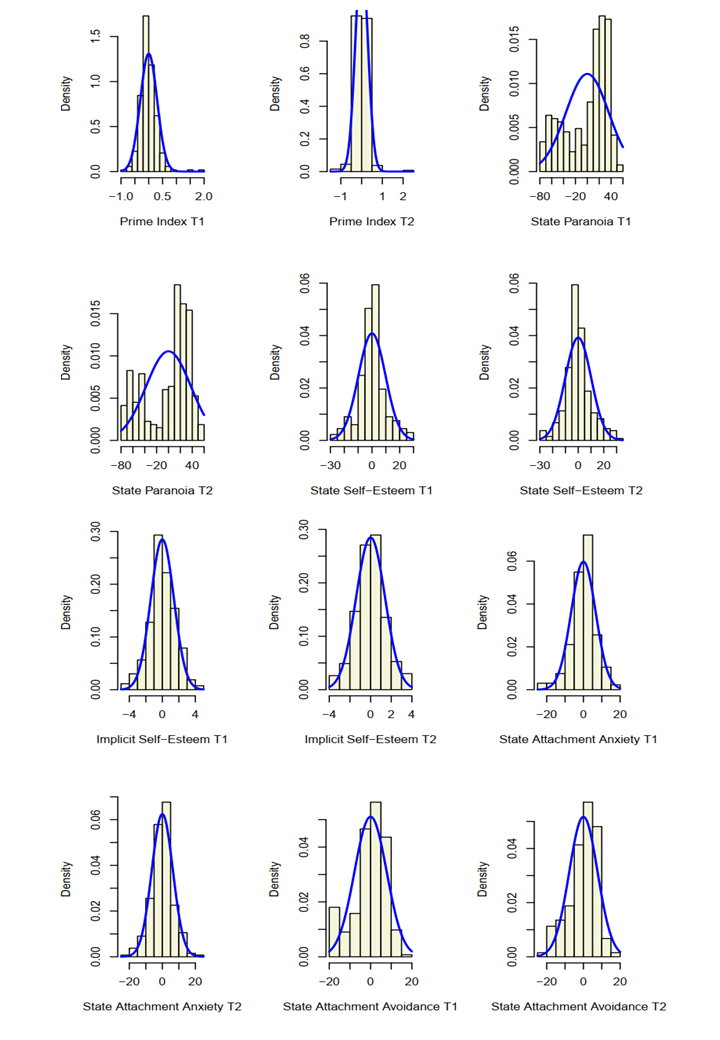
**

|  |  |  |  |  |  |  |  |  |  |  |  |  |  |  |  |
| --- | --- | --- | --- | --- | --- | --- | --- | --- | --- | --- | --- | --- | --- | --- | --- |
| **Table S3.** *Bivariate correlation between trait and state psychological variables measured at baseline.* | | | | | | | | | | | | | | | |
| Variables | | | *M* | *SD* | 1 | 2 | 3 | 4 | 5 | 6 | 7 | 8 | 9 | 10 |  |
| 1. Paranoia Trait | | | 28.96 | 6.49 | 1 | **.55^**^** | **-.31^**^** | -.10 | -.11 | .02 | **.27^**^** | **.66^**^** | **.79^**^** | -.51^**^ |  |
| 1. Negative Self-Esteem | | | 23.80 | 4.08 |  | 1 | **-.40^**^** | -.12 | .09 | .05 | .11 | **.47^**^** | **.43^**^** | **-.64^**^** |  |
| 1. Attachment Anxiety | | | .66 | 3.86 |  |  | 1 | .07 | .01 | -.02 | **-.25^**^** | **-.16^**^** | **-.19^**^** | **.33^**^** |  |
| 1. Attachment Avoidant | | | .004 | 3.26 |  |  |  | 1 | .03 | .019 | .06 | **-.25^**^** | -.03 | .11 |  |
| 1. Implicit Self- Esteem (T1) | | | .42 | 1.56 |  |  |  |  | 1 | -.018 | **-.14^*^** | **-.18^**^** | **-.24^**^** | **-.14^*^** |  |
| 1. Prime Index - **β** (T1) | | | .03 | .30 |  |  |  |  |  | 1 | -.04 | .02 | .01 | .01 |  |
| 1. State Attachment Anxiety (T1) | | | 30.25 | 6.69 |  |  |  |  |  |  | 1 | **.16^**^** | **.25^**^** | **-.20^**^** |  |
| 1. State Attachment Avoidant (T1) | | | 27.01 | 7.87 |  |  |  |  |  |  |  | 1 | **.68^**^** | **-.41^**^** |  |
| 1. State Paranoia (T1) | | | 93.18 | 36.06 |  |  |  |  |  |  |  |  | 1 | **-.32^**^** |  |
| 1. State Self-Esteem (T1) | | | 60.63 | 9.77 |  |  |  |  |  |  |  |  |  | 1 |  |
| *Note.*^**^ Correlation is significant at the 0.01 level (2-tailed), ^*^ is significant at the 0.05 level (2-tailed) | | | | | | | | | | | | | | | |
